# Mapping Wildfire Ignition Probability Using Sentinel 2 and LiDAR (Jerte Valley, Cáceres, Spain)

**DOI:** 10.3390/s18030826

**Published:** 2018-03-09

**Authors:** Yolanda Sánchez Sánchez, Antonio Martínez-Graña, Fernando Santos Francés, Marina Mateos Picado

**Affiliations:** 1 Department of Geology, Faculty of Sciences, Plaza de la Merced s/n, University of Salamanca, 37008 Salamanca, Spain; yolanda.ss@usal.es; 2 Department of Soil Sciences, Faculty of Environmental Sciences, Avenue Filiberto Villalobos, 119, University of Salamanca, 37007 Salamanca, Spain; fsantos@usal.es; 3 Fundation Tormes EB, Calle Toro, 37002 Salamanca, Spain; mmp@usal.es

**Keywords:** Sentinel 2, LiDAR, probability of ignition, fuel model maps, wildfire, natural hazards

## Abstract

Wildfire is a major threat to the environment, and this threat is aggravated by different climatic and socioeconomic factors. The availability of detailed, reliable mapping and periodic and immediate updates makes wildfire prevention and extinction work more effective. An analyst protocol has been generated that allows the precise updating of high-resolution thematic maps. For this protocol, images obtained through the Sentinel 2A satellite, with a return time of five days, have been merged with Light Detection and Ranging (LiDAR) data with a density of 0.5 points/m^2^ in order to obtain vegetation mapping with an accuracy of 88% (kappa = 0.86), which is then extrapolated to fuel model mapping through a decision tree. This process, which is fast and reliable, serves as a cartographic base for the later calculation of ignition-probability mapping. The generated cartography is a fundamental tool to be used in the decision making involved in the planning of preventive silvicultural treatments, extinguishing media distribution, infrastructure construction, etc.

## 1. Introduction

Wildfire constitutes one of the main threats to natural areas, being a seasonal problem of permanent character [1], which has been aggravated by factors related to climate change and conditions of socio-economic vulnerability; [2] wildfire requires the performance and organization of a series of defense structure by an administration to ensure a quick and effective response [3].

The conditions of the Mediterranean climate, which is characterized by a long summer period with very unfavorable conditions in terms of both temperature and precipitation, together with the plant formations typical of the Mediterranean [4], make wildfire a recurring phenomenon in this area which is further aggravated by man-made factors in fire causality. Because of these factors, when taking actions related to managing wildfire, a preventive policy must prevail where proactive measures in times of low and medium danger are taken, providing the environment with defense infrastructures and specific forestry actions.

In the mid-1980s, techniques based on remote sensing for the study of wildfire began to be implemented. In recent years, advances in this area have focused on the subsequent analysis of the damage caused by wildfire [5,6], the degree of damage to vegetation [7] after a wildfire [8,9], clarifying the main causes of ignition [10], evaluating the state of reforestation [11] after the passage of the fire [12], or identifying active fire hotspots [13]. On the other hand, a parallel study has used satellite imagery to classify the fuel model with medium–low precision [14].

The classification of the fuel model is made according to the characteristics of the plant masses. These data were obtained through field observations published in the National Forest Inventory, which was an expensive and lengthy process [15]. However, such information can be obtained more quickly nowadays with LiDAR (Light Detection and Ranging) data [16,17], along with other remarkable information in the classifications fuel model [18] such as the vertical continuity of the tree masses [19,20], the topography characteristics [21], and the tree canopy structure [22,23,24]. For greater precision in the mapping of fuel models, studies have been carried out that combine the information from the multispectral images with the LiDAR data, for example in the fuel models of the BEHAVE program from the United States [25], and in simulating the behaviour of wildfire [26] when satellites are not as accessible or have less temporal and spatial resolution. From the fuel model, the mapping of the intensity of the fire [27], of the CO_2_ that will be emitted [28], or of the probability of ignition occurring [29], can all be predicted by performing interpolation processes and geostatistical analysis of meteorological data and topographic characteristics [30].

In this study, remote sensing has been implemented as an analytical tool in the field of forest fires; for example, satellite images from the Sentinel 2 satellite [31] have been used to map vegetation with a resolution of 10 × 10 m. This mapping has been related to the data obtained by LiDAR [32] of the PNOA (National Plan of Aerial Orthophotography) in order to characterize the arboreal masses and topography of the study area, and later to create an analysis protocol that allows updating of this mapping to occur periodically or immediately. Additionally, it allows the creation of specialized maps (such as fuel-model and ignition-hazard maps) as a preventive measure, rather than a structural measure, in the field of forest fires in the Jerte Valley. This has, in turn, allowed the mapping of high-resolution fuel models. This geographic information science (GIS) analysis in the Jerte Valley aims to obtain a detailed, reliable, and updatable mapping of the fuel model with a minimum amount of investment.

Once the map of fuel models is obtained, the probability ignition map is also obtained, and thus the study areas are determined when priority is given to the implementation of preventive measures [33] and the centralization of the extinction resources. Fires depend on weather conditions, and therefore this index does not consider the effect of past climatic conditions, only those occurring in the present [34].

## 2. Materials and Methods

### 2.1. Study Area

**Figure 1:**
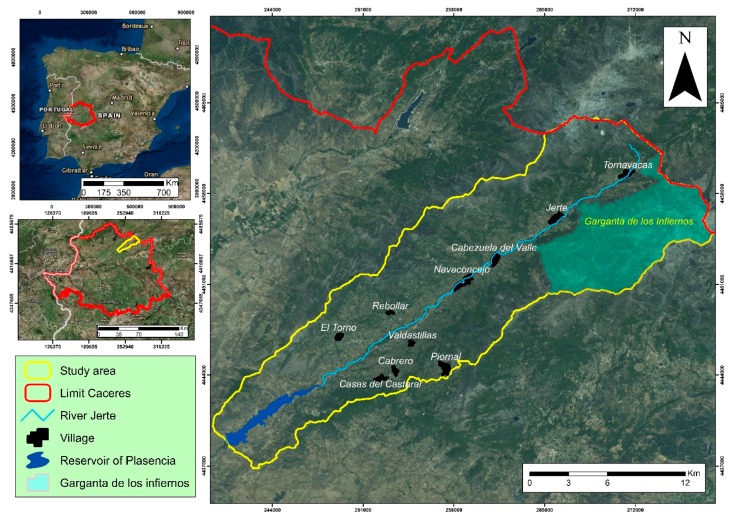
Location of the study area (the Jerte valley) within Cáceres (Spain).

The study site has an area of 376 km^2^ and corresponds to the basin slope of the Plasencia’s reservoir in the province of Cáceres. The Jerte valley (Figure 1) is located between two mountain ranges; Sierra Tormantos to the south-east, and Sierra Béjar to the north-west. The valley forms a natural corridor of communications between the southern and northern slopes.

In this valley an important protection figure, “Garganta de los Infiernos” natural reserve, is located. A morphology of steep slopes, with deep and comfortable valleys formed by runoff water, gives the landscape a natural importance.

The predominant vegetation includes orchards and irrigated arable crops (meadows), irrigated fruit trees such as cherry trees (*Prunus avium*), and insolated plots of walnuts and plums. The cherry trees occupy large areas both at the bottom of the valley and on its slopes. The grasslands are located in elevated areas, specifically heights greater than 1500 m, which usually coincide with depressions or flat ridges located between areas of scrubland and rocky outcrops. The forest area is pine forest (*Pinus pinaster*) and frequent small chestnut forests (*Castanea sativa*). In the area closest to the Plasencia’s reservoir, pasture abounds, mostly formed by holm oaks and some cork oaks [35].

The climate of the Jerte valley is characterized by an annual average precipitation that varies from 1400 mm/year in high areas, such as the Tornavacas port, to 800 mm/year in the reservoir. The annual average temperature varies from 16 °C in areas with higher altitude to about 24 °C in the lowest areas in the valley. The relative humidity varies between 40% and 50%, depending on the altitude [36].

### 2.2. Materials

#### 2.2.1. Sentinel 2

Sentinel 2 is a mission of the European Space Agency composed of the launch of two satellites—Sentinel 2A (launched in June 2015) and Sentinel 2B (launched in March 2017). The Sentinel 2 data have been used to calculate the fuel-model map. This satellite was chosen because it is the European satellite that provides the highest spatial resolution (10 m) among those that offer free services [37] and a temporary resolution of five days. The date that the photographs were taken is 16 July 2016, since this was a time when the vegetation was in full bloom and the percentage of clouds is almost nil, thus facilitating the classification of the vegetation and the ability to obtain an image with fewer distortions [38]. The satellite has 13 spectral bands (Table 1) that range from visible and near-infrared wavelengths (VNIR) to short-wave infrared (SWIR) along a 290 km orbital strip [39].

#### 2.2.2. Lidar 2 × 2

The LiDAR data has been obtained from the Spanish National Geographic Institute of the PNOA (National Plan of Aerial Orthophotography). The data of the study area were obtained in 2010 and the images (Table 2) have the calibration of the LiDAR sensor, a maximum of five returns per pulse, and a pre-classification of said returns.

#### 2.2.3. Classifications of Fuel Models

The fuel models used in Spain [40] are based on the fuel models for calculating fire behaviour used by Albini [41] to develop the nomograms published in his paper “Estimating Wildfire Behavior Effects”. There are 13 models, including 11 developed by Anderson and Brown and published by Rothermel in 1972 [42], a model for dead brush developed at the suggestion of Von Johnson, and a model for southern rough developed by Albini. These are called the “(Northern Forest Fire Laboratory) NFFL fuel models” [43] and are grouped into four categories [44]: grassland, scrubland, lush leaf under trees, and remains of cut and other forestry operations Table 3.

### 2.3. Methods

**Figure 2:**
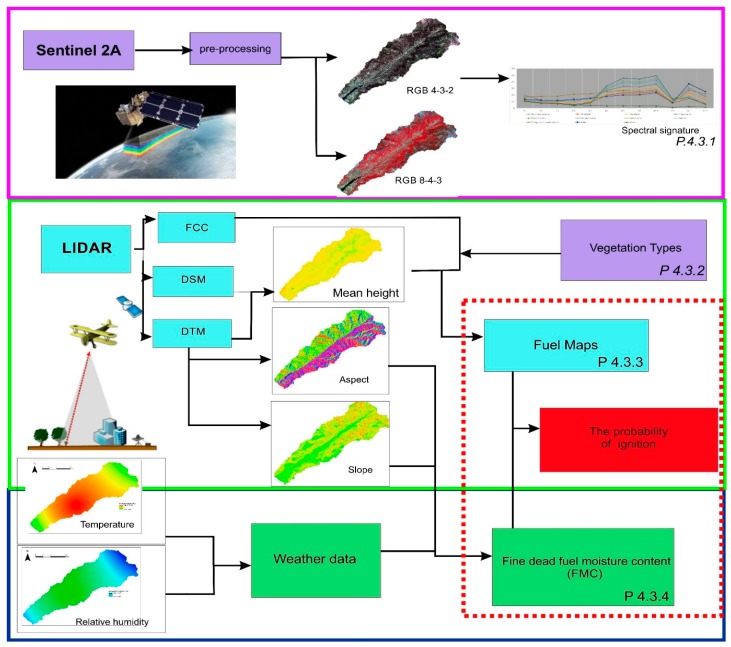
Methodological diagram.

To achieve ignition-probability mapping (Figure 2), fuel-models mapping is first needed, which is obtained using the spectral images from the Sentinel 2 satellite and from the LiDAR data. In terms of the satellite multispectral images, a supervised classification is utilized, with the samples corresponding to each one of the different vegetation categories selected in order to allow us to obtain the current vegetation mapping. Using the LiDAR data, the characteristics of the arboreal masses are obtained. On the other hand, climatic data together with the digital terrain model (DTM) are required for the calculation of fine fuel moisture. Finally, the probability of ignition is calculated.

#### 2.3.1. Vegetation Mapping

The images coming from the Sentinel 2 satellite are used to complete the vegetation mapping of the study area; however, it is necessary to carry out a series of preceding steps before beginning to work with the images, for which pre-processing has been carried out to improve their visual quality [45]. A radiometric and atmospheric calibration process [46], has been carried out using QGIS 2.18.4 Software [47] and the Semi-Automatic Classification plugin [48], which complete the calibration automatically.

**Figure 3:**
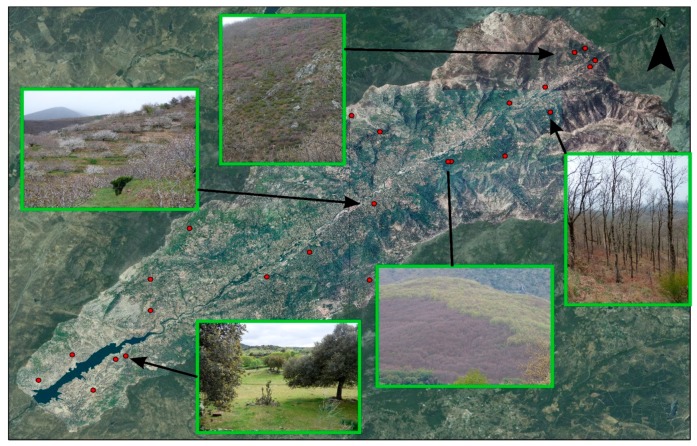
Situation of training areas.

Prior to this, the composition of the bands of natural color is determined. This image serves as a cartographic basis for the field demarcation of the training areas (Figure 3) [49] using GPS (global positioning system), which will allow the spectral signature to be obtained.

With the SCP plugin of the QGIS software, the average reflectance values of the different training areas are calculated, which allows us to make a combination of RGB (red-green-blue) bands with greater precision for the classification of the vegetation [50].

**Figure 4:**
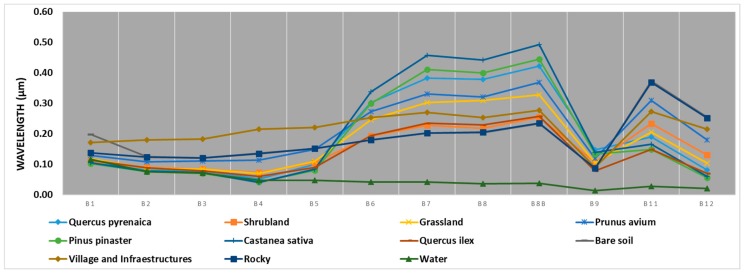
Spectral signature of the different types of vegetation and land uses in relation to wavelength (μm) by Sentinel 2 satellite bands.

Before conducting a supervised classification of the satellite images, it is useful to study the real separability of the selected categories so that they can be classified without risk of confusion. For this reason, a graph has been drawn with the spectral signature (Figure 4), which indicates the different coverage of parts of the Earth’s surface by solar radiation. This figure is very useful for the purpose of providing a first assessment of the spectral trends of each category. In addition, it identifies those spectral bands in which a peculiar behaviour with respect to the neighboring class is manifested.

Figure 4 shows the spectral signature of the different types of vegetation and land use. These have higher intervals in band 8, since the reflectance of the different parts of the Earth’s coverage are different and there will be less possibility of error in their classification. Given the resolution of 10 m, this band has been chosen for the combination of spectral bands where the classification of the vegetation will be made, since to differentiate the vegetation the best combination is that of an infrared band (B8) and two bands of the visible spectrum (B4 and B3). Consequently, the combination used for the classification of the vegetation is 8-4-3, by means of a classification supervised by the maximum likelihood method.

#### 2.3.2. Characteristics of the Arboreal Masses

In order to differentiate the arboreal masses with greater precision, a characterization of the arboreal masses was carried out via the creation of 4 rasters, with a pixel size of 2 × 2 m, using the following parameters: average height of the vegetation (Hm), global canopy cover fraction (FCCg), canopy cover fraction overstory (FCCc), and canopy cover fraction of undergrowth (FCCs). These were obtained using the LiDAR data from the study area.

**Figure 5:**
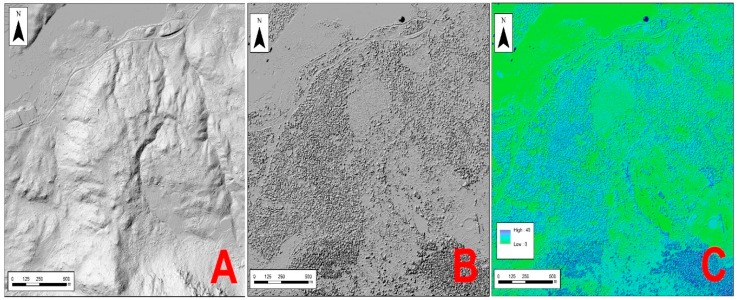
(**A**) Digital terrain model (DTM); (**B**) digital surface model (DSM); (**C**) height of the vegetation.

Firstly, the height of the vegetation was calculated [51]; a digital elevation model (DEM) (Figure 5A), by means of the returns from the soil class, and the digital surface model (DMS) (Figure 5B), using only the first returns, are used to accomplish this. Both models are filled in to avoid the possibility of pixels with no data. By subtracting using the Raster Calculator tool in the ArcMap 10.5 Software [52], the height of the vegetation is obtained (Figure 5C).

**Figure 6:**
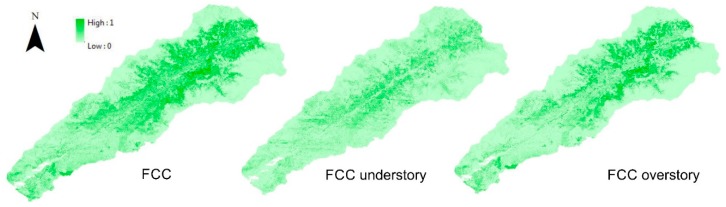
Types of canopy cover.

Then, the three types of FCC are calculated [53]. This is done by creating a LAS dataset of all returns of the soil class, and for each of the three canopy covers depending on the types of return needed (Table 4). The surface occupied by soil and vegetation is added to obtain of total surface, in this step the FCC are calculated (Figure 6).

**Figure 7:**
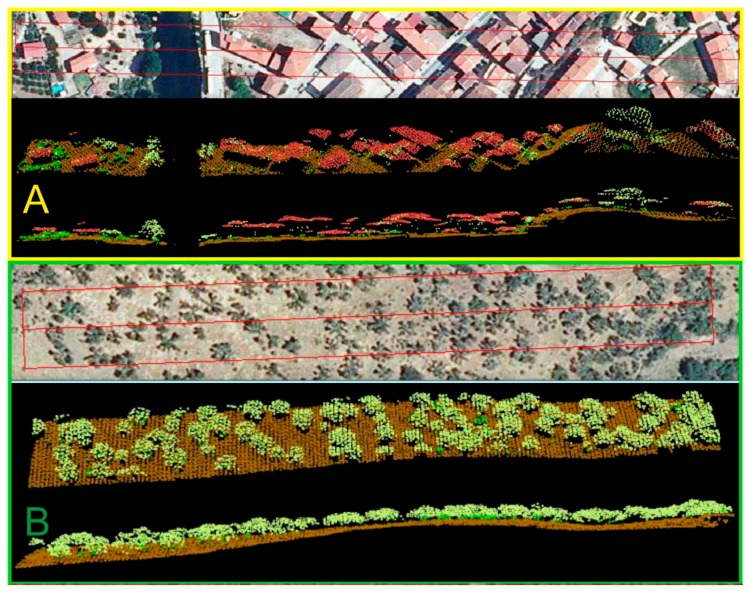

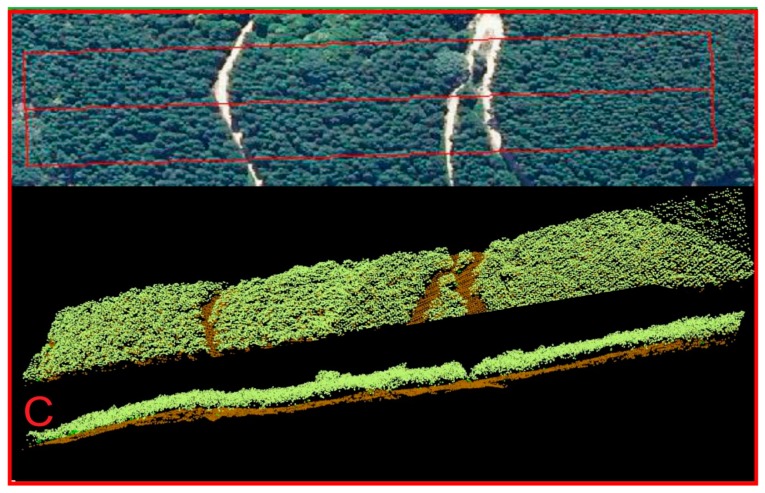
LiDAR section each section is composed by the orthophoto, The section LiDAR in oblique view and the LiDAR section in vertical view. (**A**) Model 0: Towns and infrastructure; (**B**) Model 1: FCC < 0.3; (**C**) Model 9: FCCc > 0.3 FCCs < 0.3.

In the LiDAR sections (Figure 7) we can see the different information provided by these data, such as fuel models 0 (for example, urban areas), compared with the increase of the arboreal density of model 1 compared with model 9, as well as its vertical continuity.

**Figure 8:**
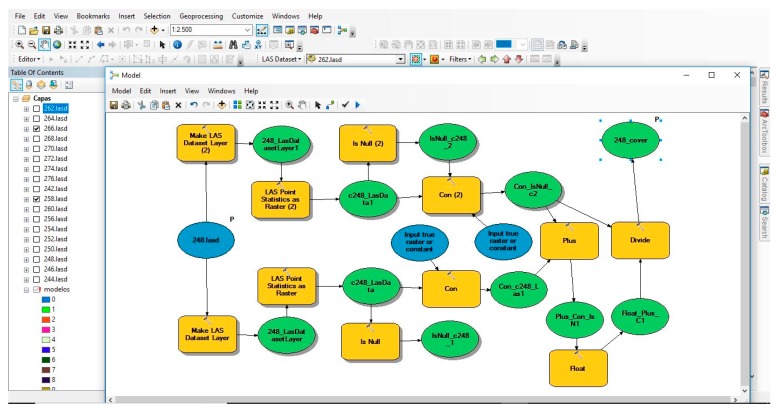
General calculation process of the FCC in Model Builder.

Automating the process by the creation of a tool with the Model Builder from ArcGIS software would allow the processing of the LiDAR data in different areas of the study area to be carried out more quickly, and this would facilitate the creation of the mapping since specialist knowledge would not be necessary and processing of LiDAR data with only GIS knowledge would be possible (Figure 8).

#### 2.3.3. Creation of Fuel-Models Mapping

The applied methodology works with the decision algorithms, using the information provided by the different rasters from the LiDAR to characterize the arboreal masses. The raster from the classification of images from Sentinel 2 [54], from vegetation mapping, allows automatic allocation of fuel models to be carried out.

First, the vegetation types (grassland, scrubland, and woodland) are differentiated by the vegetation mapping. Once the vegetation has been differentiated using the ArcGIS mathematical algorithms, the data that characterise the different arboreal masses (height of the vegetation and FCC) are extracted, since the fuel models are based on the structure and distribution of the vegetation. In order to correctly assign the types of fuel models, the following decision tree was designed (Table 5).

#### 2.3.4. Probability Ignition Mapping

The probability of ignition is the chance that a firebrand will cause an ignition when it lands on receptive fuels. It is a hazard index designed by the Instituto para la Conservación de la Naturaleza (ICONA), [55] used since 1987. This index does not take into account the state of the living vegetation, but rather estimates the moisture content of the light and dead fuels located on the forest surface from the air temperature, relative humidity, exposure, and topography.

##### Creating Trend Maps from Weather Data

From the monthly data from June to September of 2016, obtained from the meteorological stations (Table 6) located in the vicinity of the study area, the maps of average temperatures and average relative humidity were created. For this purpose, the geostatistical tool Inverse Distance Weighting (IDW) is used to generate the corresponding cartography.

**Figure 9:**
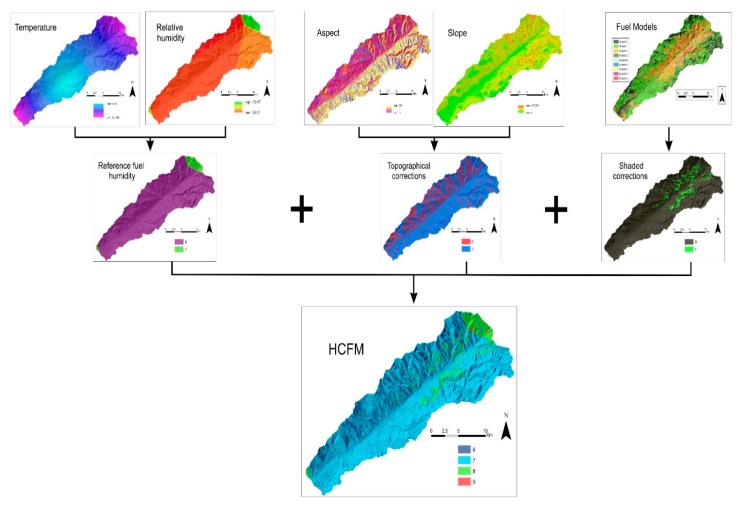
Humidity of the dead fine fuel (HCFM) calculation process.

To calculate the humidity of the dead fine fuel (HCFM) (Figure 9), from the relative humidity and temperature, the reference fuel moisture tables are used. The humidity of the dead fine fuel has to be corrected according to the topography characteristics of slope and exposure. To achieve this, the slope and orientations maps are calculated with (digital terrain model) DTM and the pertinent corrections are made with the fine dead fuel moisture tables. Fuel models 8 and 9 are assigned a value of 1 to correct for the humidity of the dead fine fuel due to shading provided by a tree. The rest are assigned a value of 0. Finally, the probability of ignition, which is the probability that a fly ash or ember falling on a dead fine fuel could ignite, is calculated using the reference fuel moisture tables and fuel model mapping [56]. Equation (1) is used for this calculation:

Reference fuel humidity + slope/orientation corrections + shading corrections = HCFM(1)

#### 2.3.5. Validation

The vegetation mapping was validated in the field, with the objectives of calculating the reliability of the same after performing the supervised classification, checking that the data obtained from the LiDAR were correct, and correcting possible anomalies.

The vegetation was verified using 50 points distributed throughout the study area in a random manner, with a minimum extension of 12 adjacent pixels belonging to the same vegetation class; 70% of these areas were used as training areas for the development of the cartography, while the remaining 30% were later used as a test to verify the accuracy of said cartography. To calculate the accuracy of the vegetation mapping, a confusion matrix was made. This is a square matrix that includes as many rows as the types of vegetation we have assigned, and as many columns as the types of vegetation to be analysed in the field. From the percentage of points corresponding to the types of vegetation assigned in relation to the total number of points, and the probability of success of the vegetation assigned in relation to the total number of points, the probability of success of the vegetation mapping was obtained. The Kappa coefficient that adjusts the effect of chance by class crossing was also calculated.

The probability ignition mapping was validated by comparing it with the historical database of wildfire corresponding to the period of climatic data used in the model.

## 3. Results

### 3.1. Vegetation Mapping

**Figure 10:**
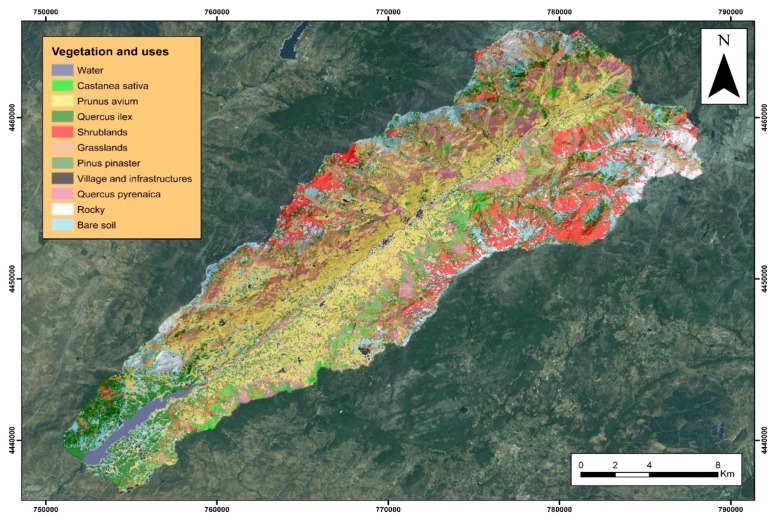
Land-use and vegetation mapping.

After performing the supervised classification (Figure 10) vegetation and land-use mapping with a high resolution (pixel of 10 × 10 m) was obtained, with which the fuel models were subsequently calculated.

Table 7 shows the error matrix of vegetation mapping after supervised classification from the nearest neighbor. The overall accuracy was 88% (Kappa index 0.86), which is considered satisfactory in terms of accuracy since it has a large number of classes with some notable similarities between them.

In general, the user’s accuracy and the producer’s accuracy in the individual classes were good since they are very close the unit except specific cases. For example, the village class have a great deal of confusion with rocky class, and the rocky class have a great deal confusion with the oak trees, because it has a large amount of bare soil, of very light colours. However, there are classes with a very high producer’s accuracy such as water, holm oaks, pines forest, chestnut forest, scubland and grassland.

### 3.2. Creation of Fuel-Models Mapping

**Figure 11:**
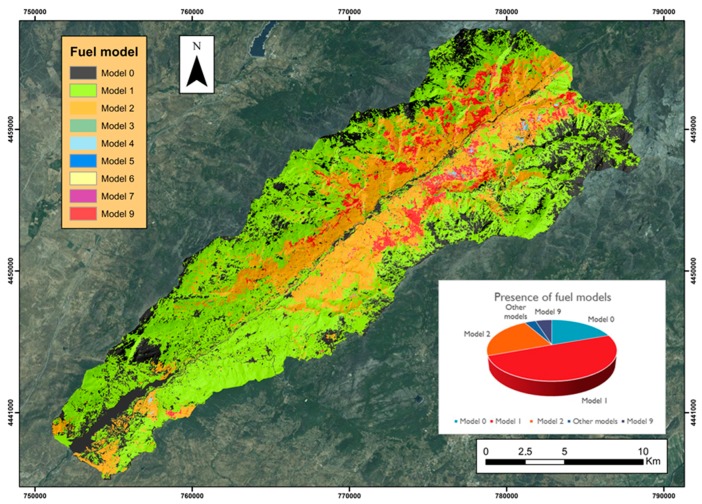
Fuel-models mapping.

Figure 11 shows the mapping generated for the classification of fuel models. The abundant fuel model in the Jerte valley is grassland (model 1 and 2) at 73%. Model 1 is located in areas of greater altitude, in the head and inferior third of the valley, while model 2 is distributed in areas of medium and low height of the upper zones and also in the vicinity of the Plasencia reservoir.

The presence of the other fuel models—scrubland (models 4–7) and little fall (model 9)—is much smaller. Among the scrubland models, it is possible to differentiate between models with a moderate load (models 5 and 6) on the southern slope, although with a very small presence, and the model with a high load (model 7), with small spots scattered in the upper half of the valley. Model 9 is located at a medium height on both slopes due to the presence of chestnut forest (*Castanea sativa*) and oak forest (*Quercus pyrenaica*).

It should be kept in mind that model 10 and the models of the remains of cut and other forestry operations (models 11–13) have not been included, since we cannot discriminate whether the remains in the undergrowth are of natural origin (pests or diseases) or due to forest holdings (forestry treatments). The assignment of the type of models associated with wooded masses would only be valid through their field verification, given the temporary nature of these disturbances.

### 3.3. Ignition-Probability Mapping

**Figure 12:**
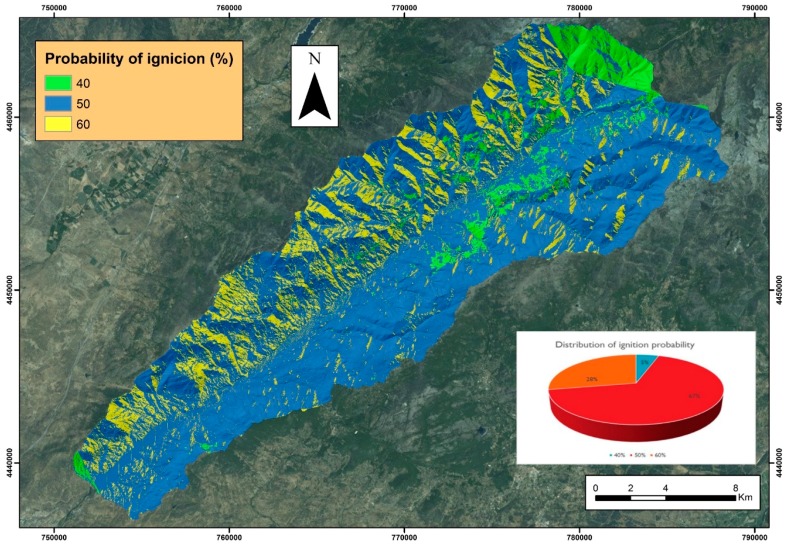
Probability of ignition mapping.

The results for ignition-probability mapping (Figure 12) show three differentiated zones with values between 40% and 60%.

The areas with the highest probability of ignition correspond to a lower relative humidity, with values around 40% and within the sun’s slopes. On the other hand, the lower ignition probabilities correspond to the presence of fuel model 9; that is, with oak forest and chestnut forest that provide shade to the underlying materials.

To validate the probability of ignition mapping, it was compared with the inventory of historical fire data in the study area. It was verified that there is indeed a relationship between the increase in the probability of ignition and the volume of outbreak fire. Thus, in the area with a 60% probability of fire, there were 2.79 fires per km^2^; in the area corresponding to a 50% probability of fire there were 2.23 fires per km^2^, and in areas of a probability of 40% there were 1.94 fires per km^2^.

The ignition-probability mapping is the base layer upon which the meteorological risk map of wildfire is built daily in the Regional Operational Centre. It is obtained by combining the probability of ignition with the wind speed, with a correction due to precipitation. The danger calculation procedure is automated on the GIS platforms, so from the presented layer of probability of ignition it is only necessary to update the daily meteorological forecasts to obtain the danger indexes.

## 4. Discussion

The results of the study support the importance of using remote sensing in wildfire prevention, since the proposed methodology allows us to obtain fuel-model mapping in a short time and with very high accuracy, as well as eliciting the probability of ignition of the different areas with accuracy and daily temporality.

Previous studies have shown that the use of images from Sentinel 2 has been very effective for the classification of both crops and forest vegetation [57]. It is considered a very promising tool for precision agriculture [58] because of its high spatial and temporal resolution, among other features. These images have been used for vegetation classification, obtaining an accuracy of 77% [59]. In the present study, satellite images have been merged with LiDAR data, increasing the accuracy to 88%. Therefore, the use of both technologies in combination is optimal for forestry studies, especially in the field of wildfire. Different studies have reached similar conclusions [60], because the LiDAR information gathers vegetation height data with great accuracy [61], while the satellite images provide information on the texture, radiation, and information of the terrain; this range of information is very useful for mapping. It has also been shown that forestry inventories are much more precise if they have been carried out by unmanned vessels rather than by field work [62], which makes this methodology faster, more economical, and more reliable.

Once vegetation mapping of a high accuracy has been obtained, and the fuel-model mapping is generated, it becomes possible to enter the data into different programs for the calculation of the behaviour of fires while also taking into account the climatic and topographic data [63].

## 5. Conclusions

The methodology described in this paper allows us to obtain fuel-model mapping with a high level of detail from structural information of the vegetation, provided by LiDAR data in combination with Sentinel 2 images. This method allows the generation of fuel-model mapping of large extensions at a very low cost, given that field work is reduced to a minimum, which allows permanent ongoing updating to occur.

Furthermore, this methodology allows us to obtain high-resolution and detailed inputs (cells of 10 × 10 m), greater than those of any mapping used prior to this, for the purpose of developing forest fire simulation software that makes it possible to anticipate with greater certainty the behaviour of potential fires.

From this work, it is concluded that the probability determined from ignition mapping allows us to generate daily maps of the danger index based on up to date meteorological forecasts with GIS scripts. This automates the process and improves the historical cartographies of fuel models and the danger of ignition, which in turn makes it possible to assess the redistribution of operational difficulties according to the mapping outputs, especially in times of low and medium danger where the devices are not fully operational.

This mapping is a preventive measure to avoid wildfires and serves as an essential tool in the planning and management of the territory, and as a decision-making tool when planning preventive forestry treatments, construction of infrastructure, etc.

## Figures and Tables

**Table 1:** Radiometric and spatial resolution of Sentinel 2.

SENTINEL-2 Radiometric and Spatial Resolutions			
Band Number	Name	Central Wavelength (nm)	Spatial Resolution (m)
1	aerosols	443	60
2	blue	490	10
3	green	560	10
4	red	665	10
5	NIR	705	20
6	NIR	740	20
7	NIR	783	20
8	NIR	842	10
8a	NIR	865	20
9	Water vapour	945	60
10	Cirrus detection	1375	60
11	SWIR	1610	20
12	SWIR	2190	20

**Table 2:** LiDAR sensor specifications.

Camera	Aerial Orthophotography
Laser spectral band	panchromatic, blue, green and red
Laser pulse density	2 points/m^2^
The pixel size	0.20 m
Flying height	Maximum 3000m
Horizontal accuracy	0.30 m
Vertical accuracy	0.20m

**Table 3:** Characteristics of fuel models.

Type	Model	Short Description
Urban area	0	Infrastructures and towns
Grasslands	1	Fine dry grass, with possible appearance of herbaceous plants covering a smaller area up to 1/3. Fuel load from 1 to 2 T/ha
	2	Fine dry grass, with clear presence of bushes and trees that cover an area of 1/3 to 2/3. Fuel load of 5 to 10 T/ha
	3	Coarse, dense, dry and high grass (>1 m). Fuel load of 4 to 6 T/ha.
Scrubland	4	Very dense or young thicket repopulate without performances. Fuel load of 25 to 35 T/ha
	5	Dense and green undergrowth less than 0.6 m high. Fuel load of 5 to 8 T/ha.
	6	Scrub older than model 5 with heights between 0.6 y 1.2 m. Fuel load of 10 to 15 T/ha.
	7	Flammable species (heath, jars) as the understory of conifers or hardwoods. Fuel load of 10 to 15 T/ha.
Lush leaf under trees	8	Dense forest of conifers and hardwoods with compact leaf litter. Fuel load of 10 to 12 T/ha.
	9	Forests with less compact leaf litter, long-leaf conifers, and broadleaved conifers. Fuel load of 7 to 9 T/ha.
	10	Dense forest with dead wood or infected forest. Fuel load of 30 to 35 T/ha.
Remains of cut and other forestry operations	11	Clear and strongly clear forest. Fuel load of 25 to 30 T/ha.
	12	Predominance of remains on the trees. Fuel load of 50 to 80 T/ha.
	13	Accumulations of thick and heavy debris covering the ground. Fuel load of 100 to 150 T/ha.

**Table 4:** Calculations of canopy cover fraction (FCC) types.

FCC_g_	Canopy cover fraction	$\frac{S_{all\;returns\;of\;vegetation}}{S_{total}}$
FCC_c_	Canopy cover fraction overstory	$\frac{S_{all\;returns\;of\;medium\;and\;high\;vegetation}}{S_{total}}$
FCC_s_	Canopy cover fraction understory	$\frac{S_{1th\;returns\;of\;high\;vegetation}}{S_{total}}$

**Table 5:** Decision tree for the classification of fuel models.

FCC < 1/3			M1
FCC 1/3–2/3			M2
FCC > 2/3	grassland		M3
	scrubland	>2 m	M4
		<0.6 m	M5
		>0.6 (0.6–1.2)	M6
		FCC overstory > 0.3 FCC understory > 0.3 0.6–2 m understory or inflammable scrubland	M7
	woodland without understory	*P. sylvestris*	M8
		FCC overstory > 0.3 FCC understory < 0.3 *Castanea sativa, Quercus* sp; *P. pinaster*	M9

**Table 6:** Stations data.

Stations	Average Temperature (°C)	Average Humidity (%)	Coordinate X (m)	Coordinate Y (m)
Losar del Barco	20.1	52.9	285,381	4,472,220
Valdeastillas	24.5	39.8	255,607	4,447,376
Gargantilla	24.0	38.9	249,777	4,458,446
Jarandilla de la Vera	24.0	41.8	274,426	4,442,377
Aldehuela del Jerte	19.1	48.1	736,412	4,433,680

**Table 7:** Error matrix of vegetation mapping.

		Reference Data											Total	User’s Accuracy (%)	Kappa
		Water	*Castanea sativa*	*Prunus avium*	*Quercus ilex*	Shrublands	Grasslands	*Pinus pinaster*	Village	*Quercus pyrenaica*	Rocky	Bare Soil			
**Classified Data**	**Water**	6	0	0	0	0	1	0	1	1	0	1	10	0.60	0
	***Castanea sativa***	0	22	0	0	0	1	0	0	1	0	0	24	0.92	0
	***Prunus avium***	0	1	102	1	2	2	0	1	1	3	7	120	0.85	0
	***Quercus ilex***	0	0	2	65	5	2	0	0	2	12	3	91	0.71	0
	**Shrublands**	0	0	0	0	59	0	0	0	0	0	0	59	1.00	0
	**Grasslands**	0	0	0	0	0	50	0	0	0	0	0	50	1.00	0
	***Pinus pinaster***	0	0	0	0	0	0	18	0	0	0	0	18	1.00	0
	**Village**	0	0	0	0	0	1	0	5	1	0	3	10	0.50	0
	***Quercus pyrenaica***	0	0	0	0	0	0	0	0	36	0	0	36	1.00	0
	**Rocky**	0	0	0	0	0	1	0	3	0	26	0	30	0.87	0
	**Bare Soil**	0	0	0	0	0	0	0	0	0	0	56	56	1.00	0
**Total**		6	23	104	66	66	58	18	10	42	41	70	504	0.00	0
**Producer’s Accuracy**		1.00	0.96	0.98	0.98	0.89	0.86	1.00	0.50	0.86	0.63	0.80	0	0.88	0
**Kappa**		0	0	0	0	0	0	0	0	0	0	0	0	0	0.86
